# *cis*-Bis(butyl­amine-κ*N*)bis­[sulfa­diazine(1−)-κ^2^ *N*,*N*′]copper(II) penta­hydrate

**DOI:** 10.1107/S1600536808026457

**Published:** 2008-08-23

**Authors:** Hümeyra Paşaoğlu, Gökhan Kaştaş, Zerrin Heren, Orhan Büyükgüngör

**Affiliations:** a Ondokuz Mayıs University, Department of Physics, Faculty of Arts and Sciences, 55139 Kurupelit Samsun, Turkey; b Ondokuz Mayıs University, Department of Chemistry, Faculty of Arts and Sciences, 55139 Kurupelit Samsun, Turkey

## Abstract

In the title compound {systematic name: *cis*-bis­[4-amino-*N*-(pyrimidin-2-yl)benzene­sulfonamidato-κ^2^ *N*,*N*′]bis­(butyl­amine-κ*N*)copper(II) penta­hydrate}, [Cu(C_10_H_9_N_4_O_2_S)_2_(C_4_H_11_N)_2_]·5H_2_O or [Cu(sdz)_2_(ba)_2_]·5H_2_O [ba is butyl­amine and sdz = sulfadiazine(1−)], the copper(II) cation is six-coordinated by four N atoms of two sulfadiazine ligands and two N atoms of butyl­amine ligands. The copper(II) ion and one of the water mol­ecules lie on twofold rotation axes. One of the butyl groups is disordered over two sites, with occupancies of 0.395 (8) and 0.605 (8). The geometry around the S atom is distorted tetra­hedral. The crystal structure involves inter­molecular N—H⋯N and N—H⋯O hydrogen bonds. N—H⋯N hydrogen bonds between sdz ligands lead to a sheet structure parallel to the *ab* plane.

## Related literature

For related structures, see: Heren *et al.* (2006 ▶); Chung *et al.* (1975 ▶).
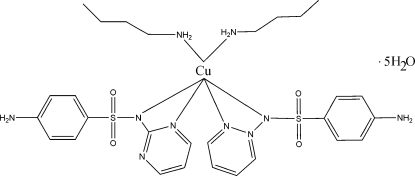


## Experimental

### 

#### Crystal data

[Cu(C_10_H_9_N_4_O_2_S)_2_(C_4_H_11_N)_2_]·5H_2_O
*M* *_r_* = 790.04
Orthorhombic, 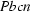

*a* = 22.623 (6) Å
*b* = 10.342 (5) Å
*c* = 16.250 (6) Å
*V* = 3802 (2) Å^3^
*Z* = 4
Mo *K*α radiation
μ = 0.74 mm^−1^
*T* = 296 K
0.34 × 0.21 × 0.19 mm

#### Data collection

Stoe IPDS2 diffractometer
Absorption correction: integration (*X-RED32*; Stoe & Cie, 2002 ▶) *T*_min_ = 0.821, *T*_max_ = 0.899
57960 measured reflections
4235 independent reflections
2098 reflections with *I* > 2σ(*I*)
*R*_int_ = 0.085

#### Refinement

*R*[*F* ^2^ > 2σ(*F* ^2^)] = 0.048
*wR*(*F* ^2^) = 0.141
*S* = 0.90
4235 reflections
234 parameters
H-atom parameters constrained
Δρ_max_ = 0.65 e Å^−3^
Δρ_min_ = −0.45 e Å^−3^

### 

Data collection: *X-AREA* (Stoe & Cie, 2002 ▶); cell refinement: *X-AREA*; data reduction: *X-RED32* (Stoe & Cie, 2002 ▶); program(s) used to solve structure: *SHELXS97* (Sheldrick, 2008 ▶); program(s) used to refine structure: *SHELXL97* (Sheldrick, 2008 ▶); molecular graphics: *ORTEP-3 for Windows* (Farrugia, 1997 ▶) and *Mercury* (Macrae *et al.*, 2006 ▶); software used to prepare material for publication: *WinGX* (Farrugia, 1999 ▶).

## Supplementary Material

Crystal structure: contains datablocks I, global. DOI: 10.1107/S1600536808026457/cf2216sup1.cif

Structure factors: contains datablocks I. DOI: 10.1107/S1600536808026457/cf2216Isup2.hkl

Additional supplementary materials:  crystallographic information; 3D view; checkCIF report

## Figures and Tables

**Table 1:** Hydrogen-bond geometry (Å, °)

*D*—H⋯*A*	*D*—H	H⋯*A*	*D*⋯*A*	*D*—H⋯*A*
N5—H5*A*⋯N2^i^	0.86	2.53	3.359 (5)	162
N4—H4*A*⋯O5^ii^	0.90	2.45	3.337 (6)	171
N4—H4*B*⋯O4^iii^	0.90	2.25	3.119 (6)	161
N5—H5*B*⋯O5^iv^	0.86	2.26	3.113 (5)	170

Symmetry codes: (i) 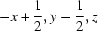; (ii) 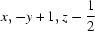; (iii) 
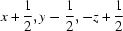
; (iv) 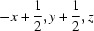.

**Fig. 1.**
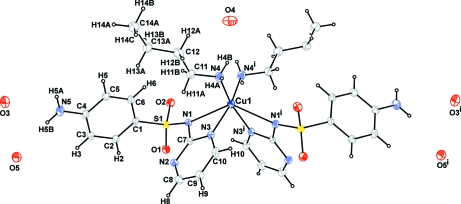
The molecular structure of (I), with atom labels and 30% probability displacement ellipsoids. Only the major part of the disordered ba ligand is included. [Symmetry code: (i) -x + 1, y, -z + 1/2.]

**Fig. 2.**
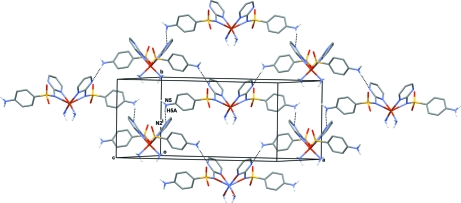
A view of the complex showing the sheet structure parallel to the ab plane. The butyl groups, water molecules and some hydrogen bonds have been omitted for clarity. Other hydrogen bonds are shown as dashed lines. [Symmetry code: (i) -x + 1/2, y - 1/2, z.]

## supplementary crystallographic information

### Comment

In the title complex (I), the copper(II) ion is six-coordinated by four N atoms of sulfadiazine ligands and two N atoms of butylammonium ligands. The copper(II) ion and one of the water molecules lie on twofold rotation axes. It is found that the Cu–N_sdz_ and Cu–N_ba_ bond distances are nearly equal. The bond angles around the S atom correspond to a distorted tetrahedral geometry. The C4–N5 bond distance and the torsion angle C1–S1–N1–C7 are comparable to those observed in related structures (Heren *et al.*, 2006; Chung *et al.*, 1975). One of the butyl groups is disordered over two sites with occupancies of 0.395 (8):0.605 (8) (see Fig. 1).

The packing of (I) is stabilized by intermolecular N—H···N and N—H···O hydrogen bonds (Table 1). The N—H···N hydrogen bond takes place between sdz ligands and it is seen that these hydrogen bonds generate a sheet structure parallel to the *ab* plane (Fig. 2). The H atoms of water molecules could not be located from a Fourier map. However, it is possible to see that water molecules are involved in hydrogen bonds with sdz and ba ligands on the basis of interatomic distances.

### Experimental

A solution of butylamine (2 mmol) in ethanol (20 ml) was added dropwise with stirring to a solution of Cu(II) sulfadiazine (1 mmol) in methanol (40 ml). The solution was heated and stirred for 3 h at 343 K and then the mixture was cooled to room temperature. The blue crystals were filtered off, washed with cold distilled water and acetone, and dried *in vacuo*. Analysis calculated: C 42.62, H 5.84, N 17.76%; found: C 43.08, H 5.72, N 18.25%.

### Refinement

One butyl group shows disorder and was modelled with two different orientations and site occupancies of 0.395 (8):0.605 (8). The H atoms of water molecules could not be located from a Fourier map. All other H atoms were placed in geometrically idealized positions with distances N—H = 0.86–0.90 Å, C—H = 0.93–0.97 Å, and were refined as riding atoms with *U*_iso_(H) = 1.2*U*_eq_(C,*N*) and *U*_iso_(H_methyl_) = 1.5*U*_eq_(C).

### Crystal data

**Table:**

[Cu(C_10_H_9_N_4_O_2_S)_2_(C_4_H_11_N)_2_]·5H_2_O	*F*_000_ = 1594
*M**_r_* = 790.04	*D*_x_ = 1.347 Mg m^−^^3^
Orthorhombic, *P**b**c**n*	Mo *K*α radiation λ = 0.71069 Å
Hall symbol: -P 2n 2ab	Cell parameters from 30097 reflections
*a* = 22.623 (6) Å	θ = 1.8–27.1º
*b* = 10.342 (5) Å	µ = 0.74 mm^−^^1^
*c* = 16.250 (6) Å	*T* = 296 K
*V* = 3802 (2) Å^3^	Prism, blue
*Z* = 4	0.34 × 0.21 × 0.19 mm

### Data collection

**Table:**

Stoe IPDS2 diffractometer	4235 independent reflections
Radiation source: fine-focus sealed tube	2098 reflections with *I* > 2σ(*I*)
Monochromator: graphite	*R*_int_ = 0.085
*T* = 296 K	θ_max_ = 27.2º
rotation method scans	θ_min_ = 1.8º
Absorption correction: integration(X-RED32; Stoe & Cie, 2002)	*h* = −28→28
*T*_min_ = 0.821, *T*_max_ = 0.900	*k* = −13→13
57960 measured reflections	*l* = −20→20

### Refinement

**Table:**

Refinement on *F*^2^	Secondary atom site location: difference Fourier map
Least-squares matrix: full	Hydrogen site location: inferred from neighbouring sites
*R*[*F*^2^ > 2σ(*F*^2^)] = 0.048	H-atom parameters constrained
*wR*(*F*^2^) = 0.141	*w* = 1/[σ^2^(*F*_o_^2^) + (0.0787*P*)^2^] where *P* = (*F*_o_^2^ + 2*F*_c_^2^)/3
*S* = 0.90	(Δ/σ)_max_ < 0.001
4235 reflections	Δρ_max_ = 0.65 e Å^−^^3^
234 parameters	Δρ_min_ = −0.45 e Å^−^^3^
Primary atom site location: structure-invariant direct methods	Extinction correction: none

### Special details

**Table:**

Geometry. All e.s.d.'s (except the e.s.d. in the dihedral angle between two l.s. planes) are estimated using the full covariance matrix. The cell e.s.d.'s are taken into account individually in the estimation of e.s.d.'s in distances, angles and torsion angles; correlations between e.s.d.'s in cell parameters are only used when they are defined by crystal symmetry. An approximate (isotropic) treatment of cell e.s.d.'s is used for estimating e.s.d.'s involving l.s. planes.
Refinement. Refinement of *F*^2^ against ALL reflections. The weighted *R*-factor *wR* and goodness of fit *S* are based on *F*^2^, conventional *R*-factors *R* are based on *F*, with *F* set to zero for negative *F*^2^. The threshold expression of *F*^2^ > σ(*F*^2^) is used only for calculating *R*-factors(gt) *etc*. and is not relevant to the choice of reflections for refinement. *R*-factors based on *F*^2^ are statistically about twice as large as those based on *F*, and *R*- factors based on ALL data will be even larger.

### Fractional atomic coordinates and isotropic or equivalent isotropic displacement parameters (Å^2^)

**Table:**

	*x*	*y*	*z*	*U*_iso_*/*U*_eq_	Occ. (<1)
C1	0.33524 (14)	0.7316 (3)	0.45323 (19)	0.0565 (8)	
C2	0.29637 (16)	0.8185 (4)	0.4879 (2)	0.0684 (9)	
H2	0.3109	0.8929	0.5129	0.082*	
C3	0.23634 (16)	0.7970 (4)	0.4860 (2)	0.0752 (10)	
H3	0.2108	0.8559	0.5107	0.090*	
C4	0.21358 (15)	0.6879 (4)	0.4473 (2)	0.0689 (9)	
C5	0.25293 (17)	0.6011 (4)	0.4128 (2)	0.0728 (10)	
H5	0.2387	0.5271	0.3870	0.087*	
C6	0.31255 (15)	0.6227 (3)	0.4160 (2)	0.0664 (9)	
H6	0.3382	0.5628	0.3927	0.080*	
C7	0.41743 (13)	0.8242 (3)	0.3032 (2)	0.0565 (8)	
C8	0.36943 (17)	0.9966 (4)	0.2474 (3)	0.0800 (11)	
H8	0.3452	1.0686	0.2536	0.096*	
C9	0.39032 (17)	0.9691 (4)	0.1702 (3)	0.0847 (12)	
H9	0.3802	1.0190	0.1247	0.102*	
C10	0.42700 (16)	0.8638 (4)	0.1637 (2)	0.0745 (10)	
H10	0.4427	0.8426	0.1125	0.089*	
C11	0.3906 (2)	0.4981 (5)	0.2216 (4)	0.124 (2)	
H11A	0.3706	0.5801	0.2136	0.149*	
H11B	0.3925	0.4841	0.2806	0.149*	
C12	0.3518 (2)	0.4000 (5)	0.1892 (4)	0.1116 (17)	
H12A	0.3740	0.3199	0.1854	0.134*	
H12B	0.3409	0.4246	0.1337	0.134*	
C13A	0.2962 (4)	0.3733 (8)	0.2362 (5)	0.088 (2)	0.605 (8)
H13A	0.2764	0.4549	0.2461	0.105*	0.605 (8)
H13B	0.3070	0.3376	0.2893	0.105*	0.605 (8)
C13B	0.2848 (5)	0.4111 (13)	0.1823 (9)	0.088 (2)	0.395 (8)
H13C	0.2709	0.4851	0.2134	0.105*	0.395 (8)
H13D	0.2733	0.4223	0.1253	0.105*	0.395 (8)
C14A	0.2505 (12)	0.277 (3)	0.1934 (18)	0.108 (4)	0.605 (8)
H14A	0.2169	0.2658	0.2286	0.162*	0.605 (8)
H14B	0.2692	0.1954	0.1841	0.162*	0.605 (8)
H14C	0.2380	0.3132	0.1418	0.162*	0.605 (8)
C14B	0.261 (2)	0.299 (4)	0.213 (3)	0.108 (4)	0.395 (8)
H14D	0.2186	0.3043	0.2115	0.162*	0.395 (8)
H14E	0.2736	0.2879	0.2693	0.162*	0.395 (8)
H14F	0.2741	0.2270	0.1810	0.162*	0.395 (8)
N1	0.43499 (11)	0.7422 (3)	0.36300 (16)	0.0586 (7)	
N2	0.38163 (12)	0.9265 (3)	0.31428 (17)	0.0635 (7)	
N3	0.44067 (12)	0.7910 (3)	0.22883 (16)	0.0596 (7)	
N4	0.44912 (14)	0.5158 (3)	0.1947 (2)	0.0896 (10)	
H4A	0.4478	0.5356	0.1408	0.108*	
H4B	0.4677	0.4391	0.1990	0.108*	
N5	0.15418 (15)	0.6661 (4)	0.4447 (2)	0.1001 (11)	
H5A	0.1407	0.5976	0.4213	0.120*	
H5B	0.1302	0.7209	0.4664	0.120*	
O1	0.42208 (10)	0.8844 (2)	0.48919 (14)	0.0690 (6)	
O2	0.43907 (10)	0.6530 (2)	0.49927 (15)	0.0731 (7)	
O3	0.02149 (14)	0.6126 (3)	0.4017 (2)	0.1193 (11)	
O4	0.0000	0.7493 (5)	0.2500	0.151 (2)	
O5	0.43200 (14)	0.3833 (3)	0.5011 (3)	0.1410 (15)	
S2	0.41156 (4)	0.75774 (8)	0.45398 (5)	0.0588 (2)	
Cu1	0.5000	0.64823 (6)	0.2500	0.0647 (2)	

### Atomic displacement parameters (Å^2^)

**Table:**

	*U*^11^	*U*^22^	*U*^33^	*U*^12^	*U*^13^	*U*^23^
C1	0.0482 (17)	0.062 (2)	0.0590 (17)	−0.0025 (16)	0.0034 (15)	0.0033 (16)
C2	0.056 (2)	0.068 (2)	0.081 (2)	−0.0050 (18)	0.0009 (18)	−0.0092 (19)
C3	0.053 (2)	0.080 (2)	0.093 (3)	0.0044 (19)	0.0133 (19)	−0.005 (2)
C4	0.051 (2)	0.078 (2)	0.078 (2)	−0.0120 (19)	−0.0005 (17)	0.007 (2)
C5	0.059 (2)	0.077 (2)	0.083 (2)	−0.013 (2)	−0.0008 (19)	−0.005 (2)
C6	0.054 (2)	0.069 (2)	0.076 (2)	−0.0018 (17)	0.0045 (17)	−0.0079 (18)
C7	0.0426 (17)	0.064 (2)	0.063 (2)	−0.0072 (16)	0.0046 (15)	0.0020 (16)
C8	0.067 (2)	0.084 (3)	0.088 (3)	0.012 (2)	−0.003 (2)	0.020 (2)
C9	0.068 (2)	0.105 (3)	0.081 (3)	0.004 (2)	−0.003 (2)	0.028 (2)
C10	0.057 (2)	0.100 (3)	0.066 (2)	−0.001 (2)	0.0069 (17)	0.007 (2)
C11	0.089 (3)	0.096 (3)	0.187 (6)	−0.029 (3)	0.050 (3)	−0.047 (4)
C12	0.080 (3)	0.108 (4)	0.146 (4)	−0.006 (3)	0.002 (3)	−0.049 (3)
C13A	0.079 (4)	0.092 (5)	0.092 (5)	−0.012 (4)	0.002 (4)	−0.018 (4)
C13B	0.079 (4)	0.092 (5)	0.092 (5)	−0.012 (4)	0.002 (4)	−0.018 (4)
C14A	0.081 (11)	0.103 (9)	0.140 (15)	−0.017 (6)	−0.014 (8)	−0.034 (8)
C14B	0.081 (11)	0.103 (9)	0.140 (15)	−0.017 (6)	−0.014 (8)	−0.034 (8)
N1	0.0480 (15)	0.0615 (16)	0.0664 (16)	0.0056 (13)	0.0059 (12)	0.0030 (14)
N2	0.0588 (17)	0.0636 (17)	0.0679 (17)	0.0086 (14)	0.0022 (14)	0.0019 (15)
N3	0.0506 (16)	0.0697 (17)	0.0585 (16)	−0.0061 (14)	0.0041 (12)	−0.0026 (14)
N4	0.070 (2)	0.087 (2)	0.112 (3)	0.0022 (18)	0.0034 (19)	−0.021 (2)
N5	0.0505 (18)	0.110 (3)	0.140 (3)	−0.0131 (19)	0.004 (2)	−0.009 (2)
O1	0.0574 (14)	0.0741 (15)	0.0755 (14)	−0.0064 (12)	−0.0044 (12)	−0.0138 (12)
O2	0.0606 (15)	0.0799 (16)	0.0787 (15)	0.0053 (13)	−0.0093 (12)	0.0243 (13)
O3	0.079 (2)	0.125 (3)	0.155 (3)	0.0017 (19)	0.018 (2)	−0.008 (2)
O4	0.174 (6)	0.091 (3)	0.188 (6)	0.000	0.053 (4)	0.000
O5	0.073 (2)	0.096 (2)	0.253 (5)	0.0007 (18)	−0.015 (3)	−0.001 (3)
S2	0.0469 (4)	0.0667 (5)	0.0629 (5)	−0.0008 (4)	−0.0020 (4)	0.0035 (4)
Cu1	0.0516 (3)	0.0697 (4)	0.0726 (4)	0.000	0.0051 (3)	0.000

### Geometric parameters (Å, °)

**Table:**

C1—C6	1.377 (5)	C12—C13B	1.524 (13)
C1—C2	1.378 (5)	C12—H12A	0.970
C1—S2	1.748 (3)	C12—H12B	0.970
C2—C3	1.377 (5)	C13A—C14A	1.592 (16)
C2—H2	0.930	C13A—H13A	0.970
C3—C4	1.391 (5)	C13A—H13B	0.970
C3—H3	0.930	C13B—C14B	1.37 (5)
C4—N5	1.363 (5)	C13B—H13C	0.970
C4—C5	1.382 (5)	C13B—H13D	0.970
C5—C6	1.368 (5)	C14A—H14A	0.960
C5—H5	0.930	C14A—H14B	0.960
C6—H6	0.930	C14A—H14C	0.960
C7—N2	1.344 (4)	C14B—H14D	0.960
C7—N1	1.351 (4)	C14B—H14E	0.960
C7—N3	1.361 (4)	C14B—H14F	0.960
C8—N2	1.334 (5)	N1—S2	1.579 (3)
C8—C9	1.371 (6)	N3—Cu1	2.025 (3)
C8—H8	0.930	N4—Cu1	2.002 (3)
C9—C10	1.373 (5)	N4—H4A	0.900
C9—H9	0.930	N4—H4B	0.900
C10—N3	1.336 (4)	N5—H5A	0.860
C10—H10	0.930	N5—H5B	0.860
C11—N4	1.407 (5)	O1—S2	1.449 (2)
C11—C12	1.441 (6)	O2—S2	1.450 (2)
C11—H11A	0.970	Cu1—N4^i^	2.002 (3)
C11—H11B	0.970	Cu1—N3^i^	2.025 (3)
C12—C13A	1.496 (9)		
			
C6—C1—C2	118.4 (3)	C12—C13A—H13B	108.3
C6—C1—S2	119.9 (3)	C14A—C13A—H13B	108.3
C2—C1—S2	121.8 (3)	H13A—C13A—H13B	107.4
C3—C2—C1	121.0 (3)	C14B—C13B—C12	107 (2)
C3—C2—H2	119.5	C14B—C13B—H13C	110.2
C1—C2—H2	119.5	C12—C13B—H13C	110.2
C2—C3—C4	120.4 (4)	C14B—C13B—H13D	110.2
C2—C3—H3	119.8	C12—C13B—H13D	110.2
C4—C3—H3	119.8	H13C—C13B—H13D	108.5
N5—C4—C5	121.0 (4)	C13A—C14A—H14A	109.5
N5—C4—C3	120.8 (4)	C13A—C14A—H14B	109.5
C5—C4—C3	118.1 (3)	H14A—C14A—H14B	109.5
C6—C5—C4	120.9 (4)	C13A—C14A—H14C	109.5
C6—C5—H5	119.6	H14A—C14A—H14C	109.5
C4—C5—H5	119.6	H14B—C14A—H14C	109.5
C5—C6—C1	121.2 (3)	C13B—C14B—H14D	109.5
C5—C6—H6	119.4	C13B—C14B—H14E	109.5
C1—C6—H6	119.4	H14D—C14B—H14E	109.5
N2—C7—N1	125.1 (3)	C13B—C14B—H14F	109.5
N2—C7—N3	123.4 (3)	H14D—C14B—H14F	109.5
N1—C7—N3	111.5 (3)	H14E—C14B—H14F	109.5
N2—C8—C9	124.2 (4)	C7—N1—S2	120.8 (2)
N2—C8—H8	117.9	C8—N2—C7	116.2 (3)
C9—C8—H8	117.9	C10—N3—C7	118.1 (3)
C8—C9—C10	116.3 (4)	C10—N3—Cu1	134.4 (2)
C8—C9—H9	121.8	C7—N3—Cu1	106.8 (2)
C10—C9—H9	121.8	C11—N4—Cu1	119.4 (3)
N3—C10—C9	121.7 (4)	C11—N4—H4A	107.5
N3—C10—H10	119.1	Cu1—N4—H4A	107.5
C9—C10—H10	119.1	C11—N4—H4B	107.5
N4—C11—C12	123.4 (4)	Cu1—N4—H4B	107.5
N4—C11—H11A	106.5	H4A—N4—H4B	107.0
C12—C11—H11A	106.5	C4—N5—H5A	120.0
N4—C11—H11B	106.5	C4—N5—H5B	120.0
C12—C11—H11B	106.5	H5A—N5—H5B	120.0
H11A—C11—H11B	106.5	O1—S2—O2	113.88 (15)
C11—C12—C13A	117.0 (5)	O1—S2—N1	113.99 (15)
C11—C12—C13B	125.3 (6)	O2—S2—N1	104.77 (15)
C11—C12—H12A	108.0	O1—S2—C1	107.76 (15)
C13A—C12—H12A	108.0	O2—S2—C1	108.16 (15)
C13B—C12—H12A	125.1	N1—S2—C1	108.02 (15)
C11—C12—H12B	108.0	N4—Cu1—N4^i^	93.7 (2)
C13A—C12—H12B	108.0	N4—Cu1—N3^i^	162.98 (13)
C13B—C12—H12B	70.1	N4^i^—Cu1—N3^i^	92.38 (13)
H12A—C12—H12B	107.3	N4—Cu1—N3	92.38 (13)
C12—C13A—C14A	116.0 (14)	N4^i^—Cu1—N3	162.98 (13)
C12—C13A—H13A	108.3	N3^i^—Cu1—N3	86.36 (16)
C14A—C13A—H13A	108.3		
			
C6—C1—C2—C3	0.5 (5)	C9—C10—N3—Cu1	−169.6 (3)
S2—C1—C2—C3	179.3 (3)	N2—C7—N3—C10	−0.2 (5)
C1—C2—C3—C4	−1.4 (6)	N1—C7—N3—C10	179.9 (3)
C2—C3—C4—N5	−179.6 (4)	N2—C7—N3—Cu1	171.8 (2)
C2—C3—C4—C5	1.4 (6)	N1—C7—N3—Cu1	−8.1 (3)
N5—C4—C5—C6	−179.5 (4)	C12—C11—N4—Cu1	−176.0 (5)
C3—C4—C5—C6	−0.5 (6)	C7—N1—S2—O1	−55.6 (3)
C4—C5—C6—C1	−0.4 (6)	C7—N1—S2—O2	179.3 (2)
C2—C1—C6—C5	0.4 (5)	C7—N1—S2—C1	64.1 (3)
S2—C1—C6—C5	−178.4 (3)	C6—C1—S2—O1	174.2 (3)
N2—C8—C9—C10	−1.4 (6)	C2—C1—S2—O1	−4.6 (3)
C8—C9—C10—N3	1.1 (6)	C6—C1—S2—O2	−62.3 (3)
N4—C11—C12—C13A	166.6 (7)	C2—C1—S2—O2	118.9 (3)
N4—C11—C12—C13B	−149.2 (9)	C6—C1—S2—N1	50.6 (3)
C11—C12—C13A—C14A	172.7 (14)	C2—C1—S2—N1	−128.2 (3)
C13B—C12—C13A—C14A	59.0 (16)	C11—N4—Cu1—N4^i^	108.9 (5)
C11—C12—C13B—C14B	−132 (2)	C11—N4—Cu1—N3^i^	−140.5 (5)
C13A—C12—C13B—C14B	−42 (2)	C11—N4—Cu1—N3	−55.2 (4)
N2—C7—N1—S2	2.7 (4)	C10—N3—Cu1—N4	−79.3 (4)
N3—C7—N1—S2	−177.4 (2)	C7—N3—Cu1—N4	110.6 (2)
C9—C8—N2—C7	0.9 (6)	C10—N3—Cu1—N4^i^	169.9 (4)
N1—C7—N2—C8	179.8 (3)	C7—N3—Cu1—N4^i^	−0.1 (5)
N3—C7—N2—C8	−0.1 (5)	C10—N3—Cu1—N3^i^	83.7 (3)
C9—C10—N3—C7	−0.4 (5)	C7—N3—Cu1—N3^i^	−86.4 (2)

Symmetry codes: (i) −*x*+1, *y*, −*z*+1/2.

### Hydrogen-bond geometry (Å, °)

**Table:**

*D*—H···*A*	*D*—H	H···*A*	*D*···*A*	*D*—H···*A*
N5—H5A···N2^ii^	0.86	2.53	3.359 (5)	162
N4—H4A···O5^iii^	0.90	2.45	3.337 (6)	171
N4—H4B···O4^iv^	0.90	2.25	3.119 (6)	161
N5—H5B···O5^v^	0.86	2.26	3.113 (5)	170

Symmetry codes: (ii) −*x*+1/2, *y*−1/2, *z*; (iii) *x*, −*y*+1, *z*−1/2; (iv) *x*+1/2, *y*−1/2, −*z*+1/2; (v) −*x*+1/2, *y*+1/2, *z*.
